# Screening of a long-term sample set reveals two *Ranavirus* lineages in British herpetofauna

**DOI:** 10.1371/journal.pone.0184768

**Published:** 2017-09-20

**Authors:** Stephen J. Price, Alexandra Wadia, Owen N. Wright, William T. M. Leung, Andrew A. Cunningham, Becki Lawson

**Affiliations:** 1 UCL Genetics Institute, Gower Street, London, United Kingdom; 2 Institute of Zoology, ZSL, Regents Park, London, United Kingdom; 3 University of York, York, United Kingdom; 4 School of Biosciences, Cardiff University, Cardiff, Wales, United Kingdom; University of South Dakota, UNITED STATES

## Abstract

Reports of severe disease outbreaks in amphibian communities in mainland Europe due to strains of the common midwife toad virus (CMTV)-like clade of *Ranavirus* are increasing and have created concern due to their considerable population impacts. In Great Britain, viruses in another clade of *Ranavirus*–frog virus 3 (FV3)-like—have caused marked declines of common frog (*Rana temporaria*) populations following likely recent virus introductions. The British public has been reporting mortality incidents to a citizen science project since 1992, with carcasses submitted for post-mortem examination, resulting in a long-term tissue archive spanning 25 years. We screened this archive for ranavirus (458 individuals from 228 incidents) using molecular methods and undertook preliminary genotyping of the ranaviruses detected. In total, ranavirus was detected in 90 individuals from 41 incidents focused in the north and south of England. The majority of detections involved common frogs (90%) but also another anuran, a caudate and a reptile. Most incidents were associated with FV3-like viruses but two, separated by 300 km and 16 years, involved CMTV-like viruses. These British CMTV-like viruses were more closely related to ranaviruses from mainland Europe than to each other and were estimated to have diverged at least 458 years ago. This evidence of a CMTV-like virus in Great Britain in 1995 represents the earliest confirmed case of a CMTV associated with amphibians and raises important questions about the history of ranavirus in Great Britain and the epidemiology of CMTV-like viruses. Despite biases present in the opportunistic sample used, this study also demonstrates the role of citizen science projects in generating resources for research and the value of maintaining long-term wildlife tissue archives.

## Introduction

Viruses in the genus *Ranavirus* present a substantial disease risk to amphibian populations in Europe [1–3]. Ranaviruses are large double-stranded DNA viruses (family *Iridoviridae*) which can cause fatal disease in a broad range of ectothermic vertebrates and which have been included on the World Organisation for Animal Health’s list of notifiable pathogens since 2009 [4–6]. Signs of disease can be severe and include systemic haemorrhage, skin ulceration and tissue necrosis [4,5]. Ranaviruses affecting amphibians (amphibian-associated ranaviruses) belong to three main groups known as *Frog virus 3* (FV3)-like, common midwife toad virus (CMTV)-like, and *Ambystoma tigrinum virus* (ATV)-like viruses. Amphibian-associated ranaviruses have a very broad host range and have caused outbreaks of disease and mortality in reptiles and fish in addition to amphibians, in both wild and captive animals [7]. Each of the major groups is widely but patchily distributed [7]. This may in part be explained by human behaviour given the presence of ranavirus in populations of invasive species as well as farmed and traded animals [8–12]. CMTV-like viruses show the most restricted geographic range, only being previously detected in mainland Europe and Asia (e.g. [1,13]).

*Ranavirus* outbreaks have been recorded with increasing frequency on the European mainland in recent years [3,11,14–18]. FV3-like viruses are present on the continent but attention has focused on the recently described CMTV-like lineage due to the severe impacts of these viruses across host species [1,3,14,19]. Novel disease outbreaks due to CMTV-like viruses in previously well-monitored amphibian populations suggest that these viruses are emerging in European amphibian populations [1,3,14,18]. Anuran and caudate hosts in Iberia have declined dramatically due to CMTV-like viruses and reptiles are known to also be susceptible to infection and fatal disease [1,3,20,21]. The emergence of these viruses therefore poses a potentially novel threat to British herpetofauna if introduced to the British Isles from mainland Europe.

Ranavirus infections in Great Britain (GB) were first confirmed in 1992 following investigation of mortality incidents affecting adult common frogs (*Rana temporaria*) in south-east England [4]. The Frog Mortality Project (FMP) was founded at this time to collate reports of amphibian mortality from citizen scientists and to facilitate the collection of samples for examination and diagnostic testing. A comparative study of global *Ranavirus* isolates from amphibians and fish suggested an introduction to GB from North America [22]. Genetic characterisation of a small sample has since revealed virus diversity consistent with multiple introductions, potentially from different sources [23], but records of ranavirus disease in GB have always been associated with FV3-like viruses [22–24]. Models of spread based on point patterns of citizen science records of common frog mortality incidents supported a combination of natural amphibian movement and repeated translocations by people as explanations of rapid ranavirus spread following introduction [23]. Persistent outbreaks of ranavirus disease have driven marked, local declines of common frogs in England [2] and, although mortality incidents are known to affect multiple species [25], experimental evidence points to a degree of host specialisation of GB ranaviruses for the common frog [26]. The presence of other hosts may even yield a dilution effect [27].

The FMP has enabled recent studies of the emergence of ranavirus disease in GB through the database of citizen science records [23,27]. The FMP has also facilitated the ongoing submission of amphibian carcasses from mortality incidents for post-mortem examination, molecular diagnostics and reporting of detection to the government’s GB Wildlife Disease Surveillance Partnership and the World Organisation for Animal Health. However, whilst there have been periods of intensive diagnostic testing for ranavirus [4,24,28], the focus has been on detection rather than molecular characterisation of the viruses involved. We therefore used the available archive of tissues from this opportunistic sample of herpetofauna of the British Isles (specimens were received from mainland GB and other islands) spanning 25 years to conduct a preliminary genetic characterisation of viruses present in light of the emerging threat posed by CMTV-like viruses and ongoing uncertainty about pathways for introduction.

## Methods

### Sampling and archiving

The Institute of Zoology (IoZ) began to build an archive of wild amphibian and reptile tissues in 1992 after becoming a founding member of the FMP, which actively solicited and received specimens involved in multiple mortality incidents following concerned reports from members of the Great British public [4]. IoZ continued wild amphibian disease surveillance through multiple research projects from the mid-1990s to 2013 during which ranavirus continued to be detected but from which frozen tissues were not systematically archived. National scanning surveillance of wild amphibians and reptiles has continued since 2013 as part of the Garden Wildlife Health project (www.gardenwildlifehealth.org), which examines specimens from both single and multiple mortality incidents.

Post-mortem examinations were conducted using standardised protocols and various tissues (including liver and kidney) were archived. A frozen tissue archive was not retained from all incidents investigated over the study period; the available archive represents a subset of a convenience sample collected through opportunistic surveillance. Nevertheless, the archive consisted of tissue samples spanning 25 years from post-larval stages of multiple native and non-native species with a wide spatial distribution. Here, we sampled this archive for extracted DNA (from liver or kidney) and for liver or kidney samples suitable for DNA extraction and screened these samples for the presence of amphibian-associated ranaviruses. The majority of carcasses were examined and tissues sampled at the time of receipt but a small number had been archived whole and not subjected to previous examination. These carcasses were defrosted, post-mortem examinations were performed and liver tissue taken. All tissue samples had been stored at -20°C since being archived.

### Molecular diagnostics

#### DNA extraction

Nucleic acid extraction from liver and kidney tissues was performed using DNeasy blood and tissue kits (Qiagen)—either 96-well plate or spin-column formats—and by following the manufacturer’s instructions. To test for possible cross-contamination, DNA extraction negative controls (consisting of extraction reagents alone and no tissue) were included in each extraction batch.

#### Ranavirus detection

All samples were initially tested for the presence of ranavirus using a probe-based quantitative PCR (qPCR) assay specific to amphibian-associated ranaviruses, which targeted a 97 base pair region of the viral Major Capsid Protein gene (MCP) [29]. Reactions were set up in 20 µL volumes—10 µL TaqMan Universal 2x PCR Master Mix (Thermofisher Scientific), 5.95 µL nuclease-free water, 1 µL of each 10 µM stocks of forward (GTCCTTTAACACGGCATACCT) and reverse (ATCGCTGGTGTTGCCTATC) primers (0.5µM final concentration), 0.05µL of 100 µM stock of VIC-labelled probe (TTATAGTAGCCTRTGCGCTTGGCC; 0.25µM final concentration), and 2 µL template DNA–and run, along with two no-template controls and appropriate standards, on 0.1 mL MicroAmp Optical 96-Well Reaction Plates (Thermofisher Scientific) sealed with MicroAmp Optical Adhesive Film (Thermofisher Scientific) [29]. Plates were run on StepOnePlus Real-Time PCR Systems (Thermofisher Scientific) with the following cycle settings: 50°C for 2 min, 95°C for 10 min, and 50 cycles of 95°C for 15s and 60°C for 30s. Samples were run in duplicate and considered positive if a sigmoidal amplification curve was present which accumulated fluorescence above the threshold in both replicates.

Positive samples by qPCR were confirmed using a conventional PCR assay which also targeted the MCP, using primers 4 and 5 from Mao et al. [30]. Reactions were run in 96-well plates in 15 µL total volumes comprising 7.5 µL GoTaq 2X colourless mastermix (Promega), 0.75 µL of each primer, 4 µL of nuclease-free water, and 2 µL of template DNA. Plates were run on the GeneAmp PCR System 9700 (Thermofisher Scientific) with the following settings: 95°C for 10mins, followed by 35 cycles of denaturation at 95°C for 45 seconds, annealing at 52°C for 45s and extension at 72°C for 45s, with a final 7min extension step at 72°C before holding at 4°C. A PCR positive control of template DNA from a cultured ranavirus isolate and a no template control (nuclease-free water) were included in each PCR run. A 5 µL aliquot of each reaction was mixed with 1 µL of 6X loading buffer spiked with GelRed^TM^ DNA-binding dye (Biotium) and run on a 1% agarose gel with 100 base pair ladder (NEB) as a size marker. PCR reactions with bands of approximately the same size as the 500 base pair marker were purified and sequenced along both strands using the forward and reverse primers (Beckman Coulter Genomics). Specimens were only considered positive for ranavirus when positive results were returned from both the qPCR and conventional PCR assays.

### Assessing relationships among ranaviruses

Sanger sequencing reads were automatically trimmed of low quality base calls at both ends and the reverse complements of ‘forward’ reads were generated using a custom Unix script. Consensus sequences were calculated for each sample from the forward and reverse strands with Merger [31]. These consensus sequences were quality checked by aligning them to their forward and reverse reads [32] and mismatches edited manually following visual inspection of the alignments [33] and chromatograms [34]. The processed sequences were combined with complete MCP coding sequences from ranavirus isolates with sequenced whole genomes (S1 Table) and aligned using Mafft v7.130b with default settings [32]. The alignment was pruned to remove redundancy among British samples due to identical sequences and trimmed to the length of the shortest sequence. The final alignment was used to construct phylogenetic trees using Bayesian (Mr. Bayes [35]; default settings used for Markov chain Monte Carlo (MCMC) analysis—500,000 generations, 4 chains, 2 runs, sample frequency = 500, and a 25% burn-in) and maximum likelihood methods (RAxML [36]; 20 maximum-likelihood trees generated on distinct starting trees, 100 bootstrap replicates calculated and annotated on the best maximum-likelihood tree). The Generalised time-reversible (GTR) model of substitution was used with both methods and rate variation among sites was modelled by discrete gamma distribution with four categories. Trees were plotted using the R package *ggtree* [37].

To further differentiate a subset of the British ranavirus isolates, five additional loci (partial coding sequences from open reading frames 13R, 58L, 59R, 81L, and 82L of CMTV from Spain [GenBank accession JQ231222]) were amplified and sequenced following the methods described by Price et al. [1]. Sequences were processed and aligned as described above and then concatenated along with the MCP alignment using Phyutility v.2.7.1 [38]. Bayesian and maximum-likelihood phylogenetic trees were constructed as above using the same reference viruses. Raw pairwise genetic distances between ranavirus strains were calculated from the concatenated alignment using the function *dist*.*dna* in the R package *ape* [39]. Divergence time estimates used a likely upper limit on the substitution rate for ranaviruses to calculate a maximum-likelihood estimate of the minimum time to the most recent common ancestor with a confidence interval (following the same method as [23]). Also, sequences of British ranaviruses were used as query sequences in homology searches of the NCBI nucleotide database using default megablast settings and searches restricted to “Ranavirus (taxid:10492)” [40].

### Temporal and spatial summaries of sampling and detection

Samples were grouped into “incidents” if they originated from the same location in the same calendar year. Data were summarised by incident, host species sampled and by virus genotype. To generate latitude and longitude data as geo-references for sample origins, OSGB36 grid references were prioritised when available and converted using UK Grid Reference Finder [41]. When grid references were not available, postcodes were converted to latitude and longitude data using GeoConvert Postcode Data [42]. In cases where specimens did not have an associated grid reference or complete postcode, approximate geo-references were generated in Google Maps by dropping a pin approximately in the centre of the region returned by the highest resolution location data available (e.g. partial postcode, town, county, isle). Maps were generated in R using the *maps* and *mapdata* packages [43]. Results of diagnostic testing were summarised in R and proportions of individuals that tested positive by species were compared with Fisher’s Exact tests.

## Results

**Fig 1:**
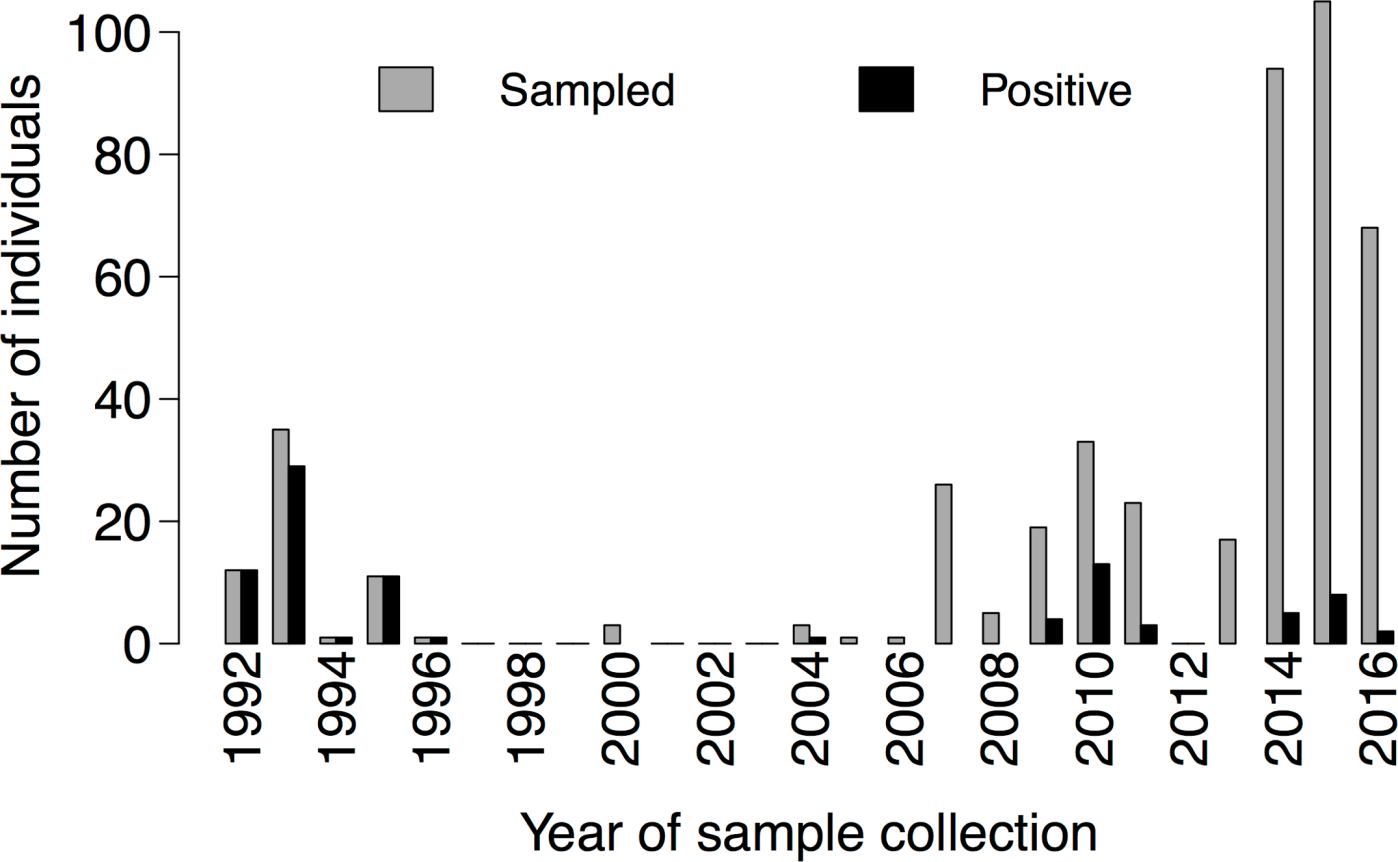
Temporal distribution of ranavirus testing and detection in amphibians and reptiles of the British Isles.

**Fig 2:**
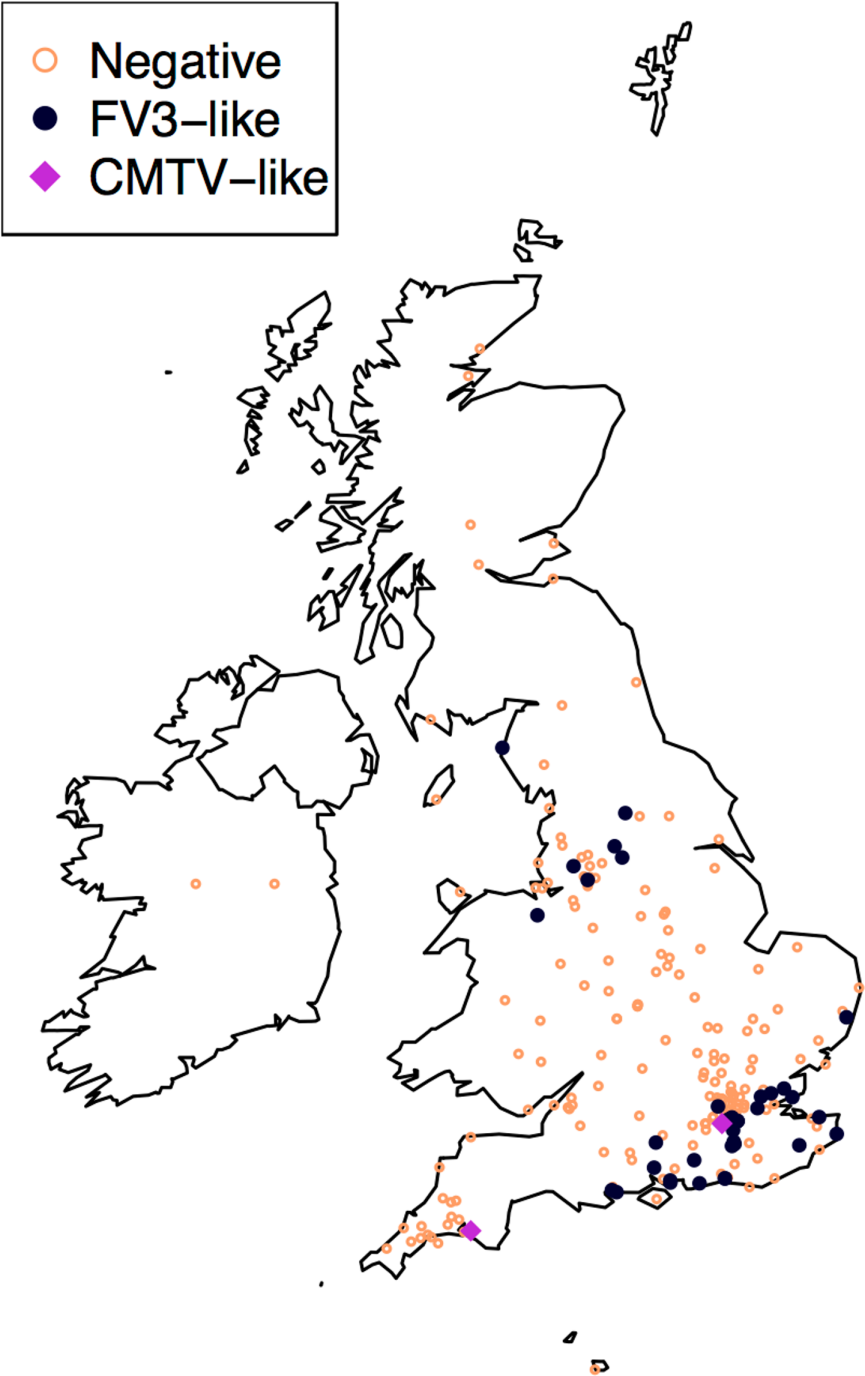
Geographical distribution of amphibian and reptilian samples tested for ranavirus in the British Isles. Colour and fill of points indicates ranavirus status (orange outline = negative) and virus genotype of positives (black circle = *Frog virus 3* (FV3)-like; purple diamond = common midwife toad virus (CMTV)-like). Due to missing data locations of 14 incidents are approximated to the highest resolution available (S1 Dataset).

A total of 458 individuals from 228 incidents (that occurred from 1992–2016 inclusive) and representing ten species (eight amphibian and two reptile) were tested for *Ranavirus* by qPCR (Table 1; S1 Dataset). Sampling was concentrated in three amphibian species; 70% of individuals tested were *Rana temporaria*, 20% were *Bufo bufo*, and 5% were *Lissotriton vulgaris*, whilst less than 1% (4/458) of samples were from reptiles. Sample counts were also aggregated in time; 58% of the samples tested were from incidents occurring between 2014 and 2016 (Fig 1). Only nine incidents (4%) involved the testing of more than a single species–eight of these involved two amphibian species and the remaining incident involved testing three species, including one reptile. Sampling was concentrated in mainland Great Britain (97% of incidents and 94% of individuals) but included five other islands of the British Isles (Fig 2; S2 Table).

**Table 1:** Sampling and results of ranavirus testing in the British Isles from 1992 to 2016 inclusive. The sample and results are summarised by species and by incident and split into incidents from which either single or multiple species were submitted and tissues were available for testing. The number of ranavirus positives (defined by positive results from both qPCR and conventional PCR assays) are given in parentheses.

Species		Single species incidents		Multiple species incidents		Total individuals
Common name	Scientific name	incidents	individuals	incidents	individuals	
Common frog	*Rana temporaria*	165 (34)	310 (73)	6 (2)	12 (8)	322 (81)
Common toad	*Bufo bufo*	39 (4)	86 (4)	2 (1)	6 (2)	92 (6)
Smooth newt	*Lissotriton vulgaris*	7 (1)	17 (1)	3 (0)	6 (0)	23 (1)
Great crested newt	*Triturus cristatus*	3 (0)	7 (0)	1 (0)	2 (0)	9 (0)
Grass snake	*Natrix natrix*	3 (0)	3 (0)	0	0	3 (0)
Palmate newt	*Lissotriton helveticus*	1 (0)	1 (0)	0	0	1 (0)
Unidentified newt	N/A	1 (0)	3 (0)	1 (1)	1 (1)	4 (1)
Slow worm	*Anguis fragilis*	0	0	1 (1)	1 (1)	1 (1)
Marsh frog	*Pelophylax ridibunda*	0	0	1 (0)	1 (0)	1 (0)
Natterjack toad	*Epidalea calamita*	0	0	1 (0)	1 (0)	1 (0)
Alpine newt	*Ichthyosaura alpestris*	0	0	1 (0)	1 (0)	1 (0)
Total		219 (39)	427 (78)	9 (2)*	31 (12)	458 (90)

*Total does not equal column sum due to counting the same incident multiple times (once for each species involved)

In total 90 individuals from 41 incidents (18% of incidents sampled) were positive for ranavirus (Table 1). The majority of positives were from *R*. *temporaria* (81/90 positive individuals); 21% of all incidents involving this species were positive. Positives were also found in *B*. *bufo* (six individuals from five incidents), *L*. *vulgaris* (one from one) and *Anguis fragilis* (one from one) plus an unidentified newt. The proportion of positive individuals to the total tested was significantly elevated in *R*. *temporaria* (Fisher’s Exact Test, p<0.001) but did not vary significantly among the remaining species tested (Fisher’s Exact Test, p = 0.2). Two out of nine incidents where multiple species were sampled were positive for ranavirus with all individuals sampled from each of these incidents being positive. *Rana temporaria* was among the positive species and was the most abundant species sampled in both of these incidents: one incident involved testing three *R*. *temporaria* individuals, one *A*. *fragilis* and an unidentified newt (i.e. a mix of anuran and caudate amphibians and a reptile) and the other involved testing five *R*. *temporaria* individuals and two *B*. *bufo*. For 32 of 41 positive incidents, ranavirus was detected in all the individuals tested. Ranavirus positive incidents were all located in two latitudinal bands in mainland GB, in the south of England and the north of England and Wales (Fig 2). Almost all the positives were from prior to 1996 or since 2009, which corresponded with sampling effort and archive availability (Fig 1).

Some or all individuals (82/90) were sequenced from all positive incidents and species (GenBank accession numbers are available in S3 Table). Clean sequences (high quality base calls across target) were obtained for 78 individuals from 38 of 41 incidents. The four poor quality sequences were all from *R*. *temporaria* and three of them corresponded to the ranavirus MCP gene but were subject to background contamination from a non-specific product which affected the quality of base calls.

**Fig 3:**
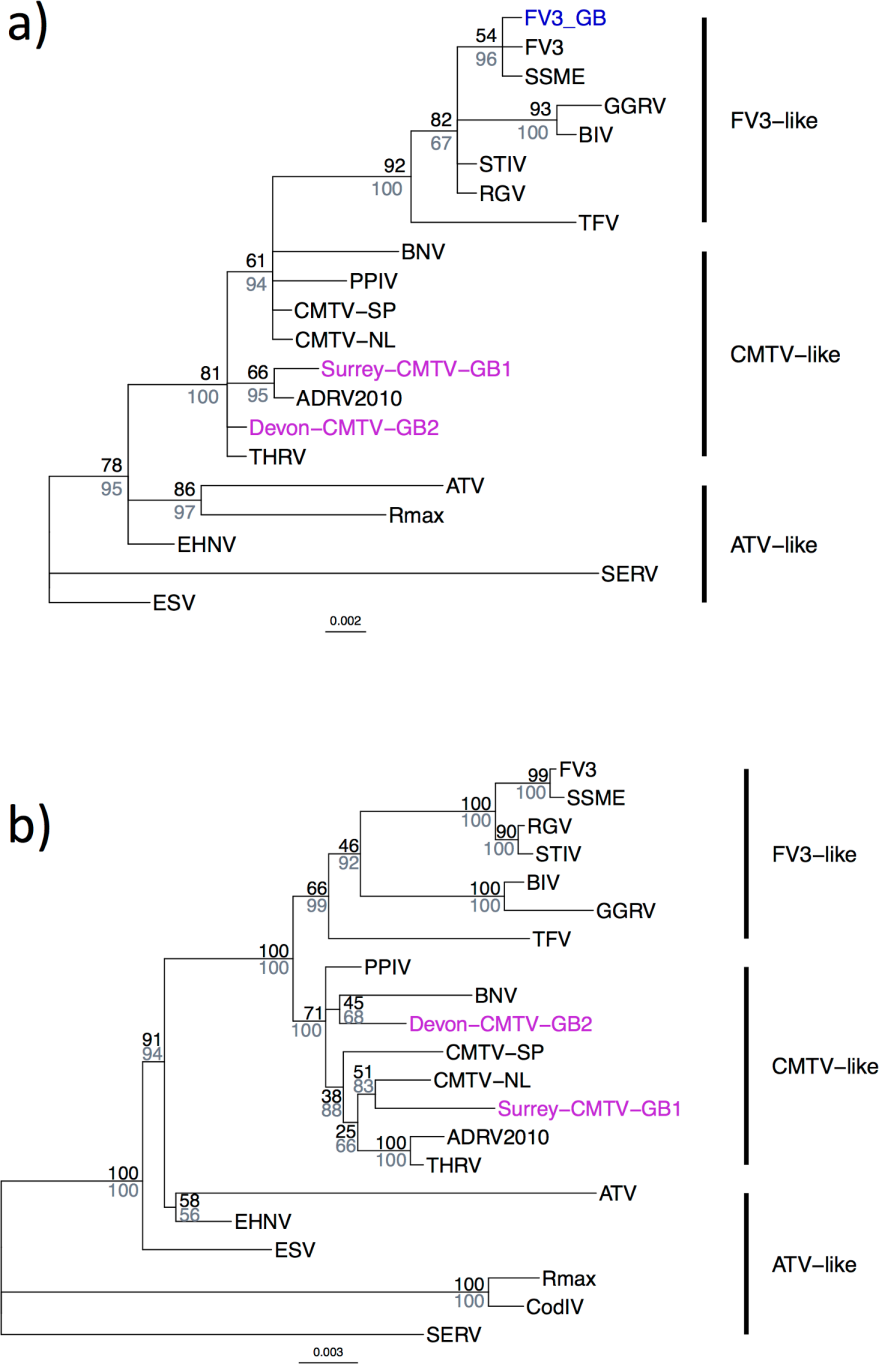
Diversity among ranaviruses detected in Great Britain in the context of amphibian-like ranavirus isolates occurring globally. (a) Phylogenetic reconstruction of *Ranavirus* relationships using a partial sequence of the major capsid protein gene (MCP); alignment length = 480 base pairs. British *Ranavirus* isolates were named according to their phylogenetic position: two British isolates belonged to the CMTV-like viruses (Surrey-CMTV-GB1 & Devon-CMTV-GB2, labelled in purple). British *Frog virus 3* (FV3)-like viruses shared 100% identity at the MCP and were grouped as ‘FV3-GB’ (blue). (b) Phylogenetic relationships based on a concatenated alignment comprising the MCP and five additional partial coding sequences (comprising Spanish common midwife toad ranavirus [CMTV-SP; GenBank accession JQ231222] open reading frames 13R, 58L, 59R, 81L, and 82L). Concatenated alignment length = 2414 base pairs. Some clades were poorly resolved and/or supported (support values less than 50). Support values at nodes were generated from posterior probabilities using Mr. Bayes (grey-blue) and summaries of 100 bootstrap replicates using RAxML (black) under a GTR model of molecular evolution and are annotated on the trees output by Mr. Bayes. Scale of branch lengths is in nucleotide substitutions per site. GenBank accession numbers for GB isolates are in S3 Table. Full isolate names, abbreviations and GenBank accession numbers for additional sequences included in the tree are given in S1 Table.

After processing the MCP sequence data and pruning to remove redundancy, a final alignment of 480 nucleotide sites and 21 taxa was obtained (18 published ranaviruses, S1 Table; three GB sequences). Phylogenetic reconstruction of *Ranavirus* relationships revealed three genotypes (two CMTV-like and one FV3-like) in GB (Fig 3A). All British FV3-like isolates were 100% identical at the MCP target region. Two incidents involved CMTV-like viruses (Fig 2). These occurrences of CMTV-like viruses were separated by approximately 300 km and 16 years. At one incident, in south-east England in 1995, Surrey-CMTV-GB1 was isolated from multiple individuals of both *R*. *temporaria* and *B*. *bufo*. At the other incident, in south-west England in 2011, Devon-CMTV-GB2 was isolated from the single *R*. *temporaria* individual sampled. The two CMTV-like isolates varied from one another at 22 sites across the 2,414 sites in the concatenated alignment of the partial MCP region and the five additional sequenced loci. The conservative estimate of the minimum time to the most recent common ancestor of Surrey-CMTV-GB1 and Devon-CMTV-GB2 was 458 years (95% confidence interval 290–681 years).

The phylogenetic trees constructed using the larger dataset (incorporating five additional markers) retained some unresolved or poorly supported clades (Fig 3B). However, all trees supported a similar topology and placed Surrey-CMTV-GB1 and Devon-CMTV-GB2 in the CMTV-like group (Fig 3). The Devon-CMTV-GB2 isolate shared 100% sequence identity at the MCP with an isolate from a captive Hermann’s tortoise (*Testudo hermanii*) from a zoo in Switzerland (accession numbers AF114154 and KP266741 [17,21]) but shared highest sequence identity across all six loci with pike-perch iridovirus (99.46%; Fig 3B; S4 Table) isolated from apparently healthy pike-perch (*Sander lucioperca*) fingerlings in Finland in 1995 [44,45]. The Surrey-CMTV-GB1 isolate was 100% identical across the partial MCP sequence to Rana esculenta virus from Italy (accession FJ358611 [46]), but among ranaviruses with whole genomes enabling comparison across all six loci, Surrey-CMTV-GB1 showed highest sequence identity to CMTV isolated in the Netherlands in 2013 (99.1%; Fig 3B; S4 Table) [47].

## Discussion

Common frog populations in Great Britain have been severely affected by *Ranavirus* for approximately three decades and mortality incidents in GB have previously been assumed to result only from infection with FV3-like viruses [2,24]. In contrast, CMTV-like viruses are emerging among amphibian populations in mainland Europe and have driven declines across a broad range of hosts [1,3]. A recent report of high sequence identity among some GB ranaviruses and Chinese giant salamander viruses [48] underscored the need for baseline data on GB ranavirus genotype to guide future surveillance and management actions. We sampled a 25-year archive of amphibian mortality incidents in the British Isles and our preliminary characterisation of the ranaviruses present revealed two strains of CMTV-like ranavirus in England in addition to 39 incidents involving FV3-like viruses in England and Wales.

The large majority of incidents in GB were associated with FV3-like viruses which was the expectation at the beginning of our study [22–24]. The British FV3-like viruses were all identical at the partial MCP sequence. However, we also discovered two CMTV-like viruses in England, occurring approximately 300 km and 16 years apart. There was nothing in the available post-mortem examination reports or accompanying information about the mortality incidents that allowed us to distinguish the two incidents with CMTV-like viruses from the incidents of FV3-like virus infection. The finding of Surrey-CMTV-GB1 in a sample from 1995 represents the oldest confirmed finding of a CMTV-like ranavirus in amphibians, occurring in the same year as the discovery of a closely related virus in pike-perch in Finland [45]. The British CMTV-like viruses were more closely related to ranaviruses from mainland Europe than to one another, based on their phylogenetic relationships and genetic distances, and a conservative estimate of the time to their most recent common ancestor was 458 years. This degree of divergence clearly precludes the possibility that these isolates diverged over the course of our sampling period. The presence of two CMTV-like viruses in GB from two incidents separated in space and time begs questions about the epidemiology of CMTV-like viruses.

The distribution of CMTV-like ranaviruses in Europe and Asia suggests two possible broad and contrasting hypotheses about their epidemiology: 1) CMTV is endemic to Europe, and widespread at the continental scale but was either unobserved until recently or has only recently emerged as a cause of severe disease due to some shift in the interaction between the pathogen and amphibian hosts, or 2) CMTV is newly emerging in Europe following introduction. The existing phylogenetic evidence, which consistently shows European CMTV-like viruses at the root of the clade and all non-European CMTV-like viruses at the tip of the clade, suggests the common ancestor of CMTV-like viruses may have originated in Europe. There is good evidence in support of multiple, recent introductions of FV3-like viruses to GB followed by rapid spread [23]. This study demonstrates that CMTV-like viruses have also been present in England for almost as long as FV3-like viruses, or perhaps longer. CMTV-like viruses may also be widespread but a less-frequent cause of the overt multiple mortality incidents that generally result from infection with FV3-like viruses. Alternatively, they may be more geographically restricted in GB than FV3-like viruses, having also been introduced recently at multiple sites but having failed to spread as successfully following introduction. The high degree of relatedness to a ranavirus isolated from apparently healthy farmed fish is of interest in the context of the latter hypothesis since it raises the possibility that fish have served as a reservoir host and in translocations.

Sequencing of a partial coding sequence of the MCP gene was useful for preliminary genotyping in that it revealed the presence of viruses from two of the major groups of *Ranavirus* in GB. However, as shown previously, this region lacked the resolution for fine-scale differentiation of ranaviruses [49,50]. The MCP sequences did not capture variation among British FV3-like viruses that has been observed previously [23]. In order to characterise the divergence among the British CMTV-like viruses, we generated data from five additional loci but relationships in the CMTV-like clade remained poorly resolved. Whole-genome sequencing would likely enable us to infer geographic and temporal changes in British FV3-like viruses and would also further characterise the two British CMTV-like viruses and their relationship to strains in mainland Europe, which will be important in understanding the risk of incursion by virulent strains such as the CMTV-like viruses found in Spain. For the time being, in light of an historic role for people in facilitating the emergence of ranavirus in GB [23], it would be sensible to adopt a precautionary principle and adhere to enhanced biosecurity measures (limiting translocations and incorporating health checks and quarantine periods for animals that are moved) to avoid future pathogen introductions.

Our findings provide further evidence that both FV3-like and CMTV-like viruses are multi-host pathogens. A single species was available for testing from most incidents but two incidents where ranavirus was detected included positive individuals from multiple species. One of the “multi-species” incidents involved anurans only and was associated with one of the CMTV-like isolates (Surrey-CMTV-GB1). The other “multi-species” incident involved the full phylogenetic breadth of our observed hosts (three common frogs [anuran], an unidentified newt [caudate] and the slow worm [*A*. *fragilis;* reptile]) and was associated with an FV3-like ranavirus. We found ranavirus associated with three amphibian species (*R*. *temporaria*, *B*. *bufo* and *L*. *vulgaris*), all identified previously as ranavirus hosts [4,24], but this is the first record of ranavirus associated with a slow worm. The lack of structured sampling makes it difficult to interpret our data on affected hosts to comment on possible preferred hosts; however most positive samples were from common frogs and the proportion of positive common frogs was higher than for other species sampled which might reflect ease of detection of common frog mortality incidents and/or the experimental evidence pointing to a degree of specialisation of FV3-like viruses for this species [26]. Further work is necessary to understand the epidemiology of ranavirus disease in slow worms and other British reptiles.

The use of the available archive, which represents a subset of an opportunistic and convenience sample, limits what is possible in respect of interpreting temporal and geographic patterns of ranavirus detection. Over the 25-year history of the archive there were several changes in effort and motivation behind carcass collection, which are likely to explain changes in the number of samples received as well as the proportion of individuals positive for ranavirus through time. For example, in the early nineties, incidents matching the signs of disease and levels of mortality typical of ranavirus mortality incidents were actively sought as part of doctoral research whereas the scope of investigations has increased more recently due to the consolidation of the FMP as part of the Garden Wildlife Health project. As for the spatial patterns, the concentration of positive ranavirus detections in two bands across England is consistent with maps based on citizen science reports from the early nineties yet visualisation of the citizen science records through time shows rapid spread across central England [23]. Unfortunately, most of our sampling in central England coincided with the recent broadening of the scope of investigations and means some targeted sampling in that region is needed to evaluate the discrepancy between the spatial distribution presented here and the predicted distribution based on citizen science records.

Although we exposed limitations in the questions that can be addressed using this type of convenience sample, we were able to generate new information on the diversity and distribution of a group of important pathogens that will be useful for management efforts in the present and research efforts in the future. Our results further highlight the considerable value of citizen science projects that encourage participation by members of the public in generating resources which enable the study of wildlife health and disease [51].

## Supporting information

S1 Dataset Sample details and screening results for every individual tested for ranavirus. Dataset includes information on host, year and location as well as results of screening and virus genotyping. All locations are approximate having been subjected to geographic masking to protect the privacy of private landowners. Details of the geographic masking are included in the file.
(XLSX)

S1 Table Details of additional ranaviruses used in phylogeny construction. Full isolate names, abbreviations used and National Center for Biotechnology Information (NCBI) accession numbers for published ranaviruses used.
(DOCX)

S2 Table Summary of sampling and ranavirus detections in the British Isles by island. (DOCX)

S3 Table GenBank accession numbers for ranaviruses from Great Britain detected in this study. nd = not done.
(DOCX)

S4 Table Pairwise raw genetic distances of common midwife toad virus (CMTV)-like viruses from Europe and Asia. Full virus names and accession numbers in S1 Table.
(DOCX)
