# Supplementary material for: Screening of a long-term sample set reveals two Ranavirus lineages in British herpetofauna
Source: PLoS One. 2017 Sep 20;12(9):e0184768. doi: 10.1371/journal.pone.0184768 (PMC5607163; doi:10.1371/journal.pone.0184768)
Supplement: S1 Table — Full isolate names, abbreviations used and National Center for Biotechnology Information (NCBI) accession numbers for published ranaviruses used. (DOCX) [file pone.0184768.s002.docx]

**S1 Table. Details of additional ranaviruses used in phylogeny construction.** Full isolate names, abbreviations used and National Center for Biotechnology Information (NCBI) accession numbers for published ranaviruses used.

| **Name** | **Abbreviation** | **Accession** | **Country of origin/isolation** |
| --- | --- | --- | --- |
| Ambystoma tigrinum virus | ATV | AY150217 | USA |
| Epizootic haematopoietic necrosis virus | EHNV | FJ433873 | Australia |
| European sheatfish virus | ESV | JQ724856 | Uncertain (Europe) |
| Andrias davidianus ranavirus isolate 2010SX | ADRV2010 | KF033124 | China |
| Testudo hermanni ranavirus isolate CH8/96 | THRV | KP266741 | Switzerland |
| Common midwife toad ranavirus isolate Pelophylax kl. esculentus/2013/NL | CMTV-NL | KP056312 | Netherlands |
| Common midwife toad ranavirus isolate Mesotriton alpestris/2008/E | CMTV-SP | JQ231222 | Spain |
| Tiger frog virus | TFV | AF389451 | China |
| German gecko ranavirus isolate 2000/99 | GGRV | KP266742 | Germany |
| Bohle iridovirus isolate BIV-ME 93/35 | BIV | KX185156 | Australia |
| Frog virus 3 isolate SSME | SSME | KJ175144 | USA |
| Frog virus 3 | FV3 | AY548484 | USA |
| Soft-shelled turtle iridovirus | STIV | EU627010 | China |
| Rana grylio iridovirus | RGV | JQ654586 | China |
| Ranavirus maximus isolate SMA15001 | Rmax | KX574343 | Denmark |
| Cod iridovirus isolate GAM14001 | CodIV | KX574342 | Denmark |
| Short-finned eel ranavirus isolate ANGA14001 | SERV | KX353311 | New Zealand |
| Pike perch iridovirus isolate SLU14001 | PPIV | KX574341 | Finland |
| Bosca’s newt virus | BNV | Various [9] | Spain |
