# Supplementary material for: Screening of a long-term sample set reveals two Ranavirus lineages in British herpetofauna
Source: PLoS One. 2017 Sep 20;12(9):e0184768. doi: 10.1371/journal.pone.0184768 (PMC5607163; doi:10.1371/journal.pone.0184768)
Supplement: S2 Table — (DOCX) [file pone.0184768.s003.docx]

**S2 Table. Summary of sampling and ranavirus detections in the British Isles by island.**

|  | **Sampled** | | **Positive** | |
| --- | --- | --- | --- | --- |
| **Island** | **incidents** | **individuals** | **incidents** | **individuals** |
| Anglesey | 1 | 8 | 0 | 0 |
| GB mainland | 221 | 431 | 41 | 90 |
| Ireland | 2 | 13 | 0 | 0 |
| Isle of Man | 1 | 1 | 0 | 0 |
| Isle of Wight | 1 | 1 | 0 | 0 |
| Jersey | 1 | 2 | 0 | 0 |
| Unknown | 1 | 2 | 0 | 0 |
