# Supplementary material for: Screening of a long-term sample set reveals two Ranavirus lineages in British herpetofauna
Source: PLoS One. 2017 Sep 20;12(9):e0184768. doi: 10.1371/journal.pone.0184768 (PMC5607163; doi:10.1371/journal.pone.0184768)
Supplement: S3 Table — nd = not done. (DOCX) [file pone.0184768.s004.docx]

**S3 Table. GenBank accession numbers for ranaviruses from Great Britain detected in this study.** nd = not done.

| Sample ID |  | Accession numbers by locus (CMTV ORF ref.) | | | | | |
| --- | --- | --- | --- | --- | --- | --- | --- |
|  |  | 13R | 16L | 58L | 59R | 81L | 82L |
| Surrey_CMTV_GB1 |  | MF411059 | MF411069 | MF411063 | MF411065 | MF411067 | MF411061 |
| Devon_CMTV_GB2 |  | MF411058 | MF411068 | MF411062 | MF411064 | MF411066 | MF411060 |
| FV3_GB |  | nd | MF411070 | nd | nd | nd | nd |
