# Supplementary material for: Screening of a long-term sample set reveals two Ranavirus lineages in British herpetofauna
Source: PLoS One. 2017 Sep 20;12(9):e0184768. doi: 10.1371/journal.pone.0184768 (PMC5607163; doi:10.1371/journal.pone.0184768)
Supplement: S4 Table — Full virus names and accession numbers in S1 Table. (DOCX) [file pone.0184768.s005.docx]

**S4 Table. Pairwise raw genetic distances of common midwife toad virus (CMTV)-like viruses from Europe and Asia.** Full virus names and accession numbers in S1 Table.

|  | CMTV-SP | ADRV  2010 | CMTV-NL | THRV | BNV | PPIV | Devon-CMTV-GB2 | Surrey-CMTV-GB1 |
| --- | --- | --- | --- | --- | --- | --- | --- | --- |
| CMTV-SP (Spain, Europe) | 0.0000 |  |  |  |  |  |  |  |
| ADRV2010 (China, Asia) | 0.0086 | 0.0000 |  |  |  |  |  |  |
| CMTV-NL (Netherlands, Europe) | 0.0081 | 0.0073 | 0.0000 |  |  |  |  |  |
| THRV (Switzerland, Europe) | 0.0077 | 0.0017 | 0.0064 | 0.0000 |  |  |  |  |
| BNV (Spain, Europe) | 0.0129 | 0.0113 | 0.0113 | 0.0113 | 0.0000 |  |  |  |
| PPIV (Finland, Europe) | 0.0075 | 0.0064 | 0.0060 | 0.0056 | 0.0086 | 0.0000 |  |  |
| Devon-CMTV-GB2 | 0.0095 | 0.0073 | 0.0077 | 0.0064 | 0.0100 | 0.0054 | 0.0000 |  |
| Surrey-CMTV-GB1 | 0.0120 | 0.0094 | 0.0090 | 0.0094 | 0.0134 | 0.0096 | 0.0091 | 0.0000 |
